# Update on possible animal sources for COVID‐19 in humans

**DOI:** 10.1111/xen.12621

**Published:** 2020-06-17

**Authors:** Tanja Opriessnig, Yao‐Wei Huang

**Affiliations:** ^1^ The Roslin Institute and The Royal (Dick) School of Veterinary Studies University of Edinburgh Midlothian UK; ^2^ Department of Veterinary Diagnostic and Production Animal Medicine College of Veterinary Medicine Iowa State University Ames IA USA; ^3^ Institute of Preventive Veterinary Medicine College of Animal Sciences Zhejiang University Hangzhou China

Since our commentary on the likelihood of pigs transmitting severe acute respiratory syndrome coronavirus 2 (SARS‐CoV‐2) to humans published in March 2020,^1^ the World Health Organization (WHO) has upgraded SARS‐CoV‐2 infection to a global pandemic (https://www.who.int/dg/speeches/detail/who‐director‐general‐s‐opening‐remarks‐at‐the‐media‐briefing‐on‐covid‐19‐‐‐11‐march‐2020) and the virus has spread to all continents with direct impact in most countries. Updated SARS‐CoV‐2 data in humans are provided in Table 1. This synopsis summarizes the latest findings on animal sources that could pose a risk for human SARS‐CoV‐2 infection and hence may be important during xenotransplantation.

**TABLE 1:** Facts on high pathogenic human CoVs

Virus	Time of circulation	Laboratory confirmed cases	Deaths	Case fatality rate%	Country distribution
SARS‐CoV^a^	2002‐2003	8096	774	9.6	26
MERS‐CoV^b^	2012‐ongoing	2494	853	35	27
SARS‐CoV‐2^c^	2019‐ongoing	4 248 389	294 046	14.4	Global pandemic

Note

Situation report 115, 14 May 2020.

^a^ Source: https://www.who.int/csr/sars/country/table2004_04_21/en/.

^b^ Source:https://www.who.int/emergencies/mers‐cov/en/

^c^ Source:https://www.who.int/docs/default‐source/coronaviruse/situation‐reports/20200512‐covid‐19‐sitrep‐113.pdf?sfvrsn=feac3b6d_2.

The novel SARS‐CoV‐2 was initially observed with severe lung disease designated as coronavirus disease 2019 (COVID‐19) in a cluster of patients in Wuhan, Hubei Province in China during December 2019.^2^ Coronavirus species typically cause respiratory and gastrointestinal sickness in both humans and animals.^3^ It is recognized that SARS‐CoV‐2 can be transmitted through aerosols and direct or indirect contact.^4^, ^5^, ^6^ However, the role of animals in human infections is less clear. One of the earliest published studies investigating the ability of SARS‐CoV‐2 to replicate in various animal species found that the virus does not infect farm animals including pigs, chickens, and ducks.^7^ In support of these early results, an ongoing study conducted at the Friedrich Loeffler Institute in Germany further confirmed that pigs and chickens are not susceptible to intranasal infection with SARS‐CoV‐2 (https://promedmail.org/promed‐post/?id=7196506). Results from experimental infection trials are summarized in Table 2. Furthermore, a large Chinese study investigated naturally occurring SARS‐CoV‐2 infection in various animal species by examining antibody levels and included serum samples from 187 pigs, 107 cattle, 133 sheep, 18 horses, 153 chickens, and 154 ducks which all tested negative.

**TABLE 2:** Outcomes of SARS‐CoV‐2 infection studies in different animal species

Species	Number used	Infection/viral shedding	Respiratory Lesions	Disease	Transmission	Seroconversion	Reference
Pigs	5	‐	‐	‐	‐	0/5	^7^
	9	‐	‐	‐	‐		^a^
Chickens	5	‐	‐	‐	‐	0/5	^7^
	17	‐	‐	‐	‐		^a^
Ducks	5	‐	‐	‐	‐	0/5	^7^
Dogs	5	1/5	‐	‐	‐	2/4	^7^
Cats							
6‐9 mo	7	7/7	‐	‐	1/3	3/3	^7^
2‐3 mo	7	7/7	Yes	Yes	1/3	3/3	
4‐5 mo	6	3/3	‐	‐	3/3		^11^
Ferrets	18	10/10	Yes	Yes	Yes	6/6	^7^
	9	Yes	Yes	Yes	Yes	Yes	^a^
Bats	9	Yes	‐	‐	‐		^a^

^a^ Source: https://promedmail.org/promed‐post/?id=7196506.

It appears that ferrets, cats and to some degree also dogs are permissive to SARS‐CoV‐2 infection. In ferrets, experimental infection resulted in virus replication in the upper respiratory tract for up to 8 days without clinical signs or mortality.^7^ These findings were essentially confirmed by other groups who also established that naïve ferrets can be infected via contact exposure (https://promedmail.org/promed‐post/?id=7196506) or airborne transmission.^9^, ^10^ Furthermore, minks with naturally acquired SARS‐CoV‐2 infection have been identified (Table 3) confirming that the *Mustelidae* family, which includes ferrets, minks but also weasels, badgers, and otters, appears susceptible to SARS‐CoV‐2 infection.

**TABLE 3:** Documented naturally acquired SARS‐CoV‐2 infections in different animal species

Family	Species	Date	Location	Clinical signs	SARS‐CoV‐2 test results					Comments	Reference
					*RNA*		*Antibody*		*Virus isolation*		
*Felidae*	Domestic cat	18‐Mar	Belgium	Respiratory signs Vomiting Diarrhea	Positive (feces and vomit)		ND		ND	COVID‐19 household Cat recovered after 9 d	https://www.oie.int/fileadmin/Home/eng/Our_scientific_expertise/docs/pdf/COV‐19/Belgium_28.03.20.pdf
		02‐Apr	Hong Kong	None	Positive (oral, nasal and rectal swabs)		ND		ND	COVID‐19 household	https://promedmail.org/promed‐post/?id=7175340
		22‐Apr	New York, USA	Sneezing Ocular discharge	Positive		Positive		ND	COVID‐19 household	https://www.oie.int/wahis_2/public/wahid.php/Reviewreport/Review?reportid=34086
					Positive		Positive		ND	Outdoor access	
		01‐May	France	Mild respiratory and digestive signs	Positive (rectal swab)		ND		ND	COVID‐19 household 1/2 cats in the household affected	https://promedmail.org/promed‐post/?id=20200501.7289409
		08‐May	Spain	Respiratory signs	Positive (nasal cavity, enteric lymph node)		ND		ND	COVID‐19 household	https://english.elpais.com/society/2020‐05‐08/spain‐records‐its‐first‐case‐of‐a‐cat‐with‐coronavirus.html
	Tiger	27‐Mar	Bronx Zoo New York, USA	Dry cough Wheezing	Positive		ND		ND	Infection assumed by asymptotic zoo employee; clinical signs in five tigers and three lions; testing was done for one tiger and one lion	https://www.oie.int/wahis_2/public/wahid.php/Reviewreport/Review?page_refer=MapFullEventReport&reportid=33885
	Lion				Positive		ND		ND		https://www.oie.int/wahis_2/public/wahid.php/Reviewreport/Review?reportid=34054
*Canidae*	Dog	26‐Feb	Hong Kong	None	Positive (nasal swabs on 26‐Feb, 28‐Feb, 2‐Mar, 5‐Mar, and 9‐Mar).		Positive		Negative	COVID‐19 household Pomeranian dog, 17 y old	https://www.oie.int/wahis_2/public/wahid.php/Reviewreport/Review?reportid=33762,^14^
		17‐Mar	Hong Kong	None	Positive (nasal and oral swabs on 18‐Mar, 19‐Mar; rectal swab on 18‐Mar)		Positive		Positive	COVID‐19 household German Shepherd, 2.5 y old; 1/2 dogs infected	https://www.oie.int/wahis_2/public/wahid.php/Reviewreport/Review?reportid=33892, ^14^
		29‐Apr	North Carolina, USA	Mild cough	Positive		ND		ND	COVID‐19 household Pug	https://edition.cnn.com/2020/04/28/us/coronavirus‐us‐pug‐wellness‐trnd/index.html
*Mustelidae*	Mink	23‐Apr	The Netherlands	Gastrointestinal and respiratory signs Increased mortality	Positive		ND		ND	Mink farm 1 Two employees with COVID‐19 signs	https://www.oie.int/fileadmin/Home/eng/Our_scientific_expertise/docs/pdf/COV‐19/OIE_SARS_CoV%202_infection_of_mink_in_the_Netherlands_26April2020.pdf
		25‐Apr		Pneumonia problems Increased mortality	Positive		ND		ND	Mink farm 2 One employee with COVID‐19 signs	

ND = Not done.

Evidence that pets may be susceptible to SARS‐CoV‐2 arose first via an experimental infection trial.^7^ Specifically, 2‐6 months old cats were intranasally inoculated with SARS‐CoV‐2 and viral RNA was detected in the respiratory tract 6 days post‐infection. When the cats were placed in contact with uninfected cats, SARS‐CoV‐2 transmission occurred after 3 days and antibodies against SARS‐CoV‐2 were detected in infected and exposed cats (Table 2).^7^ Similar findings were reproduced in US cats recently.^11^ Additional accumulated evidence during a seroprevalence study further supports that SARS‐CoV‐2 is indeed capable of entering the feline population.^12^ A total of 145 cat serum samples collected from pet hospitals and animal shelters in Wuhan before and during the COVID‐19 outbreak were tested. Approximately 13.7% (15/102) of the samples collected during the outbreak were found positive by three assays while 39 samples collected prior to the COVID‐19 outbreak were all negative.^12^ Furthermore, several domestic cats, lions, and tigers with naturally acquired SARS‐CoV‐2 infection have been identified (Table 3). The susceptibility of dogs to SARS‐CoV‐2 has also been investigated. After experimental infection they may become infected at a low level with limited transmission.^7^ Natural infection, as evidenced by the presence of antibodies, SARS‐CoV‐2 RNA or both, has been identified in selected dogs in close contact with COVID‐19 patients (Table 3). A recent French study, which investigated nine cats and 12 dogs in close contact with a cluster of COVID‐19 patients, was unable to detect evidence of SARS‐CoV‐2 infection in any of the animals.^13^ Finally, the Chinese serosurveillance study that investigated farm animals also tested serum samples from 487 dogs and 87 cats collected between November 2019 and March 2020.^8^ All samples were negative for SARS‐CoV‐2 antibodies,^8^ in accordance with the French study.^13^

In summary, since SARS‐CoV‐2 emerged in the human population towards the end of 2019, it has been spreading at a high rate. There is strong evidence that SARS‐CoV‐2 from COVID‐19 infected humans can spillover to animal species within the families *Mustelidae*, *Felinae,* and *Caninae*. Infections are frequently subclinical but occasional clinical signs can be observed (Table 3). Based on available serological surveys, these are likely localized rare events; the true extent of human‐to‐animal infections requires further investigations. Moreover, animal‐to‐human SARS‐CoV‐2 infection as well as natural animal‐to‐animal transmission has yet to be confirmed and none of the species considered to be susceptible to the virus at this point are presently used for xenotransplantation. Despite not being affected by SARS‐CoV‐2 directly, pigs are being used to test novel SARS‐CoV‐2 vaccines for possible human use (https://www.pirbright.ac.uk/news/2020/03/pirbright‐begins‐testing‐new‐coronavirus‐vaccines‐animals‐help‐combat‐covid‐19).

## Funding information

Biotechnology and Biological Sciences Research Council (BBSRC), University of Edinburgh, Roslin Institute, BBS/E/D/20002173 and BBS/E/D/20002174. Specific research fund for COVID‐19, National Natural Science Foundation of China, 32041003.
